# Transgene Expression and Transposition Efficiency of Two-Component Sleeping Beauty Transposon Vector Systems Utilizing Plasmid or mRNA Encoding the Transposase

**DOI:** 10.1007/s12033-022-00642-6

**Published:** 2022-12-22

**Authors:** Natalie Tschorn, Yasemin van Heuvel, Jörn Stitz

**Affiliations:** 1 Research Group Medical Biotechnology & Bioengineering, Faculty of Applied Natural Sciences, TH Köln–University of Applied Sciences, Campusplatz 1, 51376 Leverkusen, Germany; 2 Institute of Technical Chemistry, Leibniz University Hannover, Callinstraße, 530167 Hannover, Germany

**Keywords:** Transposon vector, *Sleeping Beauty*, mRNA transfection, Transposition, Transgene expression, Producer cell line

## Abstract

The use of two-component transposon plasmid vector systems, namely, a transposase construct and a donor vector carrying the gene of interest (GOI) can accelerate the development of recombinant cell lines. However, the undesired stable transfection of the transposase construct and the sustained expression of the enzyme can cause genetic instability due to the re-mobilization of the previously transposed donor vectors. Using a *Sleeping Beauty*-derived vector system, we established three recombinant cell pools and demonstrate stable integration of the transposase construct and sustained expression of the transposase over a period of 48 days. To provide an alternative approach, transcripts of the transposase gene were generated in vitro and co-transfected with donor vector plasmid at different ratios and mediating high GOI copy number integrations and expression levels. We anticipate that the use of transposase mRNA will foster further improvements in future cell line development processes.

## Introduction

Transposon vector technology can be used to mediate highly efficient stable gene transfer allowing for the rapid establishment of transgenic cells and is thus utilized in gene therapeutic strategies as well as the development of cell lines for the production of biologics [1–4]. Two-component plasmid vector systems are most frequently used for non-therapeutic applications derived from *Sleeping Beauty* and *PiggyBac* encompassing a transposase encoding vector and a separate donor vector containing the expression cassette with the gene of interest (GOI) flanked by the inverted terminal repeats (ITRs) [5, 6]. The expressed transposase enzyme recognizes specific sequences in the ITRs and subsequently cuts out the ITR-flanked expression cassette from the donor vector plasmid and integrates the linear DNA fragment into the target cell genome. This event called transposition thus facilitates stable gene integration via a cut and paste-mechanism [7, 8]. Transposition mediated by *Sleeping Beauty*-derived transposon vectors can mediate the integration of GOIs at high copy numbers per genome and enable sustained expression [9]. When the expression of the GOI in a transposon vector is coupled to a selectable reporter gene such as antibiotic resistance genes, highly productive cell pools can be established in a couple of days and weeks. This approach circumvents the time-consuming establishment and screening of cell clones to reach high productivity [10–12].

The optimal ratio of donor vector and transposase vector is crucial for efficient transposition. A phenomenon called overproduction inhibition (OPI) caused by larger amounts of transposase enzyme appears to decrease transposition activity [13, 14]. Moreover, *Sleeping Beauty* vector-mediated transposition efficiencies vary in different cell lines and types from a range of donor species [10, 11]. This requires the optimization of transfection protocols and the careful titration of donor to transposase construct ratio to obtain high transgenic rates and GOI copy numbers per cell as a result of transposition [13, 15–17]. However, as plasmids are transfected, also stable transfection events occur, namely, the unintended integration of the donor vector including the bacteria-derived backbone and the transposase construct. Especially the undesirable integration of the transposase gene and sustained expression of the transposase enzyme can mediate the mobilization of transposed donor vector cassettes resulting in either the reintegration into new *loci* or the degradation of the mobilized DNA fragments [18]. This genetic instability in the target cell line can thus lead to a loss of productivity.

Consequently, we here established three recombinant cell pools using co-transfection of donor and transposase plasmids and subsequent selection pressure and examined transposase gene integration and expression over a period of 48 days. To circumvent the use of transposase encoding plasmids, the *SB100x* transposase gene of *Sleeping Beauty* was transcribed in vitro into mRNA as previously described by Bire et al. [19], who established this methodology for *PiggyBac*-derived vectors. Human HEK293 cells were either co-transfected with a constant amount of donor vector and the *SB100x* transcripts in different ratios or, alternatively, with an optimized quantity of transposase plasmid construct. Recombinant cell pools were established and analyzed for transgene expression levels and vector copy numbers resulting for transposition and stable transfection, respectively.

## Materials and Methods

### Cells

Adherent human embryonic kidney HEK293 cells (ATCC CRL-3216) were grown in high glucose (4.5 g/L) Dulbecco's modified Eagle's medium (DMEM) supplemented with 2 mM l-Glutamine and 10% fetal bovine serum (FBS); Gibco, Dreieich, Germany). Cells were expanded at 37 °C in a humidified atmosphere at 5% CO_2_. For passaging, cells were detached using 1 mM Ethylenediaminetetraacetic acid (EDTA) in phosphate-buffered saline (PBS). Prior to seeding, cell count was determined using an automated cell counter (fluidlab R-300; Anvajo, Dresden, Germany).

### Plasmids

The donor vector SB-EGFP-IpW and transposase expression plasmid CMV-SB100x were previously described by Berg et al. [11, 20]*.* Donor vector SB-VEGF-IpW was constructed by linearizing SB-EGFP-IpW upon restriction using the enzymes *EcoRV* and *XbaI* removing the enhanced green fluorescence protein (EGFP) encoding fragment and inserting instead an accordingly digested human codon-optimized sequence encoding human vascular endothelial growth factor (VEGF; Genscript, Piscataway Township, NJ, USA).

### Transfection and Selection of HEK293 Cells

HEK293 cells were transfected using TransIT®-LT1 transfection reagent (Mirus, Madison, WI, USA) according to manufacturer´s instructions. On the day of transfection, 5 × 10^5^ cells were seeded in 2 mL per well using six-well plates (Nunc, Wiesbaden, Germany). Cells were co-transfected in triplicate with 2.5 µg of SB-VEGF-IpW and 250 ng of transposase vector CMV-SB100x corresponding to a ratio of 1:1/10. Two days post transfection, cell pools were subjected to a selection pressure of 0.5 µg/mL of puromycin (InvivoGen, Toulouse, France), which was increased stepwise to a final concentration of 10 µg/mL over a period of 48 days.

### Detection of Transposase Coding Sequence in Genomic DNA

Genomic DNA of recombinant cell pools was harvested using the EchoLUTION Cell Culture DNA Kit (BioEcho, Cologne, Germany). Equivalents of 10,000 genomes (60 ng) of genomic DNA originating from transfected HEK293 cells and naïve cells serving as a negative control were used to perform genomic PCR using the Q5® High-Fidelity DNA Polymerase following the manufacturer’s instructions (NEB, Ipswich, MA, USA). 0.001 pg of the transposase expression plasmid (equivalent to 10^2^ copies) served as a positive control. In parallel, serial dilutions of transposase expression plasmid mixed with 60 ng genomic DNA of naïve HEK293 cells were prepared to serve as a reference standard. Using the oligonucleotides gDNA_SB100x-for: 5′-CTAGCGTGCAGACAATCGTGAGGAAGTACAAG-3′ and gDNA_SB100x-rev: 5′-TGGCCCTCGCAGACAGCGAATTAATTGAATTC-3′ generated amplicons of a size of 436 bp. PCR was performed employing an initial denaturation step of 30 s at 98 °C, followed by 35 cycles of denaturation for 10 s at 98 °C, annealing for 15 s at 63 °C and elongation for 15 s at 72 °C, and a final extension step for 2 min at 72 °C.

### Detection of SB100x Transcripts

RNA of transfected HEK293 cells was harvested using the RNeasy Mini Kit (Qiagen, Hilden, Germany) according to manufacturer’s instructions. Concentration of purified RNA was determined utilizing a NanoQuant Plate™ Reader (Tecan Trading AG, Männedorf, Switzerland). Purified RNA (1 µg) was subsequently reverse transcribed to cDNA using the LunaScript® RT SuperMix Kit (NEB, Ipswich, MA USA). For transposase gene detection, PCR was performed with 2 µL of cDNA solution serving as a template and oligonucleotides SB100x-for: 5′-ACAGGACATTCTGGAGAAACGTGCTGTGG-3′ and SB100x-rev: 5′- TTCTTCAGCTCGGCCCACAGATTCTCG-3′. PCR was conducted with the aforementioned PCR program. As an internal reaction control, the glyceraldehyde-3-phosphate dehydrogenase (GAPDH)-specific primers GAPDH cDNA for: 5′-CGGAGTCAACGGATTTGGTC-3′ and GAPDH cDNA rev: 5′-TGGGTGTCGCTGTTGAAGTC-3′ were used amplifying 800 bp long DNA fragments.

### SB100x-mRNA Synthesis and Transfection into HEK293 cells

The *SB100x* transposase coding sequence [20] was inserted into IVTRup (a gift from Ángel Raya (Addgene plasmid #101362)). The vector was linearized by restriction using the enzyme *EcoRV*. The Gibson cloning primers: Gibson-SB100x-for: 5′-GGCTGGGCTGGTACCTGTGCCCGGTGTCAGGCCAGCTGTAGTTGCAGCGGTCAGCTGCCGCTCTCTGGCCCTGTGCGAACTGCTGTGCCAGGTGCACCCTGGGGGACCAGGCTGCCTGGGCTTCCTGGAACTGGTGAAGCTGCCGCCACTTCCTCTATGCTCTCTCCAGCAGGCAATTCTGGGTAAACGATCTTCATTTGCCTATAAAGCTGCACAGCTCACAGGCCTTGGACCGTTTCTGCCCCAGCCCCAGCATTGGCCCTTTGGACAGACTCTGAAACCGTGCGCAGAACGCACCCTGTCATTACAAATGACTCCTGGAGGCAGTCCCCGGGGGCCTGGCAGGAGCACCTGTGTTTCTGTGGGGTCTGAAAATGACAGACCAATCGCTTGGGCCCGGGAGGCGGAGGTTGCAGTGGGCCGAGATCGAGACATTGCCCTCCAGCC-3′ and Gibson-SB100x rev: 5′-AGGGCATGGCCAGAAGGCAAGCCCCGCAGAAGGCAGCGATATCTTAGTATTTGGTAGCGTTTCCTTTGAACTGCTTCACC-3′ were used to amplify the *SB100x* coding sequence followed by the insertion into the linearized vector to generate the plasmid IVTRup-SB100x. The plasmid was used as a template for subsequent PCR amplification employing the oligonucleotides mRNA T7 Promoter for: 5′-GAATTGTAATACGACTCACTATAGGGCGAATTGG-3′ and mRNA Poly(T)-3′UTR rev: 5′-TTTTTTTTTTTTTTTTTTTTTTTTTTTTTT TTTTTTTTTTTTTTTTTTTTTTTTTCTAGACCCTCAC-3′. The resulting cDNA was used for in vitro transcription (IVT) of SB100x-mRNA employing HiScribe™ T7 ARCA mRNA Kit (NEB, Ipswich, MA, USA) according to the manufacturer’s instructions. To remove template DNA, 2 units of DNase I were added to each reaction tube containing the mRNA for 15 min at 37 °C. After DNase I treatment, mRNA was purified using the Monarch® RNA Cleanup Kit (NEB, Ipswich, MA, USA). RNA concentration and purity were determined using a NanoQuant Plate™ Reader (Tecan Trading AG, Männedorf, Switzerland). Aliquots were stored at − 80 °C [21].

Prior to transfection, HEK293 cells were detached using 1 mM PBS/EDTA and seeded at 8 × 10^5^ cells per well into six-well plates. Different amounts of SB100x-mRNA were co-transfected with a constant amount of 1.5 µg of donor plasmid SB-EGFP-IpW. Cells were transfected with 40 kDa linear polyethylenimine transfection reagent (PEI MAX®; Polysciences Inc., Warrington, PA,USA) at a mass ratio of 1:4 (DNA:PEI) in duplicate. Reaction tubes containing the donor vector or SB100x-mRNA, respectively, were prepared separately. In addition, co-transfection of the donor vector and the transposase encoding plasmid CMV-SB100x at a ratio of 1:1/10 was conducted. Cells transfected only with the donor vector served as controls. To ensure comparable transfection efficiencies, fill-up plasmid DNA of pUC18 was added to the reaction tubes to ensure constant total amounts of nucleic acids. Two days post transfection, 1 × 10^5^ cells were transferred to 10 cm dishes and 2 µg/mL of puromycin was added to the cultivation media. Cells were cultured as described in the materials and methods section for 16 days at constant selection pressure.

### Flow Cytometric Analysis of EGFP Expression

Recombinant HEK293 cell pools and naïve HEK293 cells serving as negative controls were detached and washed twice with PBS. Cells were resuspended in 1 mL Fluorescence Activated Cell Sorting (FACS)-buffer containing 1% fetal calf serum (FCS) in PBS/1 mM EDTA for subsequent flow cytometric analysis (S3e; BioRad, Hercules, CA, USA). The mean fluorescence intensity (MFI) mediated by EGFP expression (EGFP-MFI) was determined using the flow cytometry analysis software FlowJo (BD Biosciences; Franklin Lakes, NJ, USA).

### Determination of Vector Copy Numbers

Genomic DNA of recombinant cell pools was isolated using the EchoLUTION Cell Culture DNA Kit (BioEcho, Cologne, Germany) according to the manufacturer's instructions. Primer/probes for the detection of Woodchuck Hepatitis virus Posttranscriptional Regulatory Element (WPRE) (WPRE for: 5′-GAGGAGTTGTGGCCCGTTGT-3′; WPRE rev: 5′-TGACAGGTGGTGGCAATGCC-3′; WPRE probe: FAM-CTGTGTTTGCTGACGCAAC-BHQ1), Ampicillin (Amp for: 5′-CAGCAATAAACCAGCCAGCC-3′; Amp rev: 5′-ACTTACTCTAGCTTCCCGGC-3′; Amp probe: FAM-GCAACTTTATCCGCCTCCATC-BHQ1) and Polypyrimidine Tract Binding Protein 2 gene (PTBP2) as an internal reference (PTBP2 for: 5′-TCTCCATTCCCTATGTTCATGC-3′; PTBP2 rev: 5′-GTTCCCGCAGAATGGTGAGGTG-3′; PTBP2 probe: JOE-ATGTTCCTCGGACCAACTTG- BHQ1) [22] were utilized for Taqman® quantitative PCR employing the StepOne™ Real-Time PCR System (Thermo Scientific, Waltham, MA, USA). DNA-free samples and genomic DNA of naïve HEK293 cells served as negative controls. Serial dilutions of the reference VCN-standard plasmid containing WPRE, ampicillin resistance gene (Amp^R^), and PTBP2 sequences were used for standardization. Details of the cloning strategy are available upon reasonable request. Statistical significance was determined using the GraphPad Prism software (GraphPad software; San Diego; CA; USA) utilizing the 1-way-ANOVA test.

## Results

### Detection of the SB100x Gene in Genomic DNA of Transgenic HEK293 Cells

**Fig. 1:**
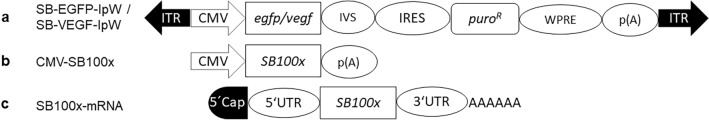
Schematic illustration of the genetic elements of transposon and transposase vector constructs. (a) A CMV promoter/enhancer element (P_CMV_) drives expression of the donor vectors and (b) the plasmid-based helper vector construct. The expression cassettes of the donor plasmids encompass the reporter gene enhanced green fluorescent protein (e*gfp)* or vascular endothelial growth factor (*vegf*) followed by an synthetic intron (IVS) and an internal ribosome entry side (IRES). Thus, the puromycin resistance gene (*puro*^*R*^) allows also for the selection of cells expressing of the other reporter gene and is followed by the woodchuck hepatitis posttranscriptional regulatory element (WPRE) and a polyadenylation signal (p(A)) of the bovine growth hormone (bGH). The expression cassettes of the the donor vectors are flanked by inverted terminal repeats (ITRs) of *Sleeping Beauty* (SB). The transposase construct CMV-SB100x encodes the human codon-optimized transposase variant *SB100x*. (c) The SB100x-mRNA comprises the transposase coding sequence and is flanked by untranslated regions (UTRs). The 5′Cap stabilizes the mRNA and protects it from degradation

**Fig. 2:**
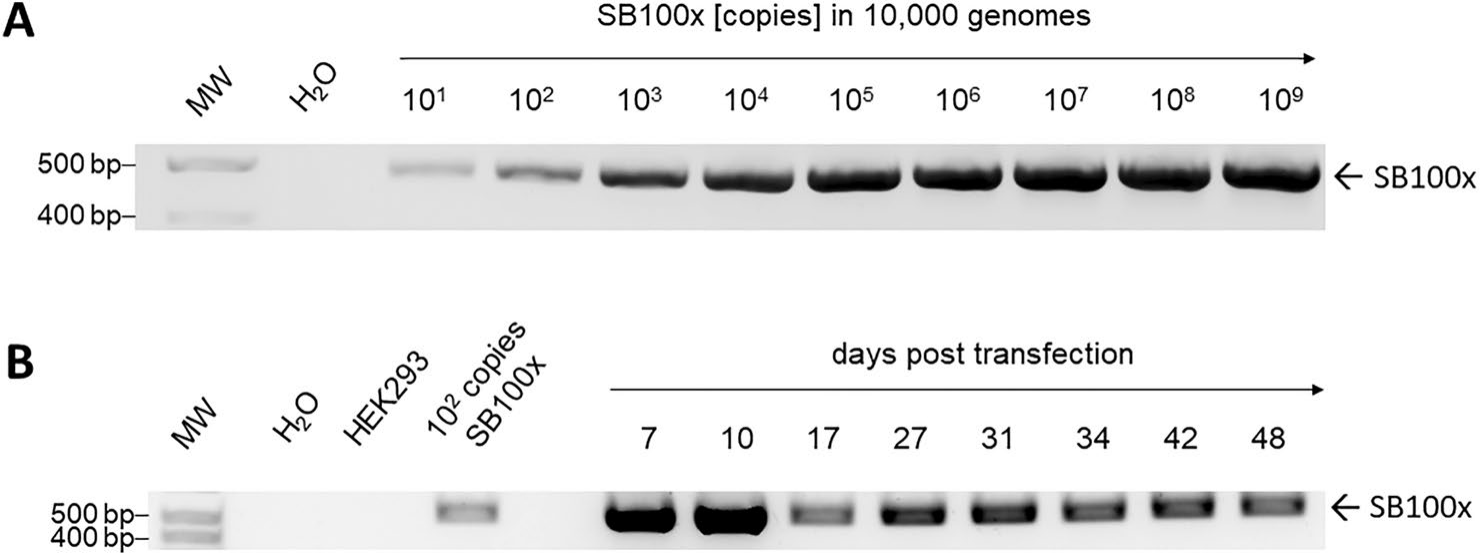
Detection of *SB100x* coding sequences in genomic DNA. **A** Different amounts of plasmid CMV-SB100x were mixed with 60 ng genomic DNA of naïve HEK293 cells equivalent to 10,000 genomes to determine the sensitivity of detection. A PCR reaction mixture without plasmid DNA template served as a negative control (H_2_O). **B** Exemplarily, one representative data set of the detection of *SB100x* gene in genomic DNA of recombinant HEK293 cell pools performed in triplicate is shown. Genomic DNA was isolated from selected cell pools upon co-transfection of donor vector SB-VEGF-IpW and transposase expression construct CMV-SB100x at different days post transfection to investigate transposase gene integration. 0.001 pg of the transposase vector mixed with 60 ng genomic DNA of naïve HEK293 cells equivalent to 10,000 genomes served as a positive control (10^2^ copies). Genomic DNA of naïve HEK293 cells and template-free samples (H_2_O) served as negative controls

Recombinant cell pools were established upon co-transfection with the donor vector SB-VEGF-IpW and transposase vector CMV-SB100x illustrated in Fig. 1 and expanded for 48 days under escalating selection pressure reaching a final puromycin concentration of 10 µg/mL at day 38. To monitor integration of the *SB100x* gene into cell genomes, genomic DNA of recombinant cell pools was isolated at different time points of cultivation. Genomic DNA of naïve HEK293 cells and a template-free sample (H_2_O) served as negative controls. PCR was conducted using *SB100x*-specific primers generating amplicons with a size of 436 bp. In parallel, serial dilutions of the transposase expression plasmid were mixed with 60 ng of genomic DNA of naïve HEK293 cells equivalent to 10,000 genomes to assess PCR sensitivity. As shown in Fig. 2 and with a sensitivity of 10 copies in 1000 genomes, *SB100x* genes were detectable throughout the entire cultivation and selection period of 48 days post transfection. Signal intensities, and thus the *SB100x* gene frequency, decreased from seven to 48 days post transfection indicating yet *SB100x* gene copy numbers of > 10^2^ and < 10^3^ integrated in 10,000 genomes.

### Detection of SB100x Transcripts in Recombinant HEK293 Cells

**Fig. 3:**
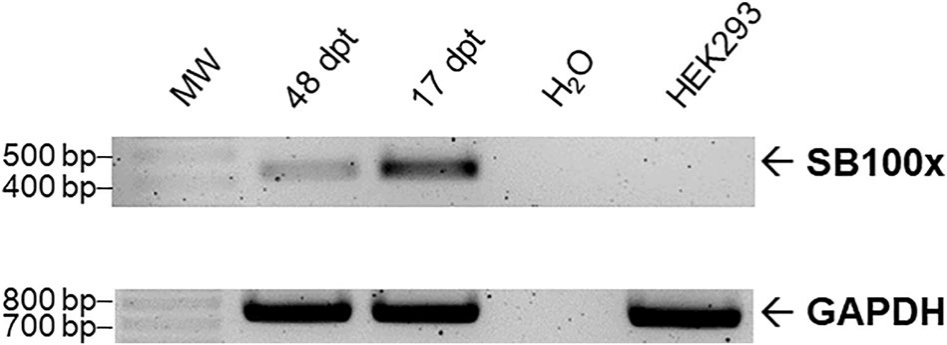
Detection of *SB100x* transcripts in HEK293 cell pools. RNA of selected cell pools was isolated 17 and 48 days post transfection (dpt) and served as templates for cDNA synthesis. Equivalent volumes of synthesized cDNA were used for the amplification of DNA fragments of the *SB100x* coding sequences. GADPH amplification served as an internal and positive control. RNA isolated from naïve HEK293 cells and template-free samples (H_2_O) served as negative controls. Data shown are representative results of experiments conducted in triplicate

To assess whether integrated transposase genes also mediated the expression of transcripts, RNA of recombinant cell pools was isolated 17 and 48 days post transfection. RNA was subsequently transcribed into cDNA and PCR was conducted employing *SB100x*-specific oligonucleotides. As an internal reference, primers specific for the amplification of GAPDH were used to ensure that similar amounts of RNA templates were applied. As shown in Fig. 3, transposase transcription was readily detected 17 and 48 days post transfection. Amplicon signal intensity decreased from day 17 to day 48, while GAPDH amplification remained constant. This demonstrated the sustained expression of transposase resulting from the unintended stable integration of the respective expression construct.

### Detection of SB100x Transcripts in Recombinant HEK293 Cells Using mRNA for Transposition

**Fig. 4:**
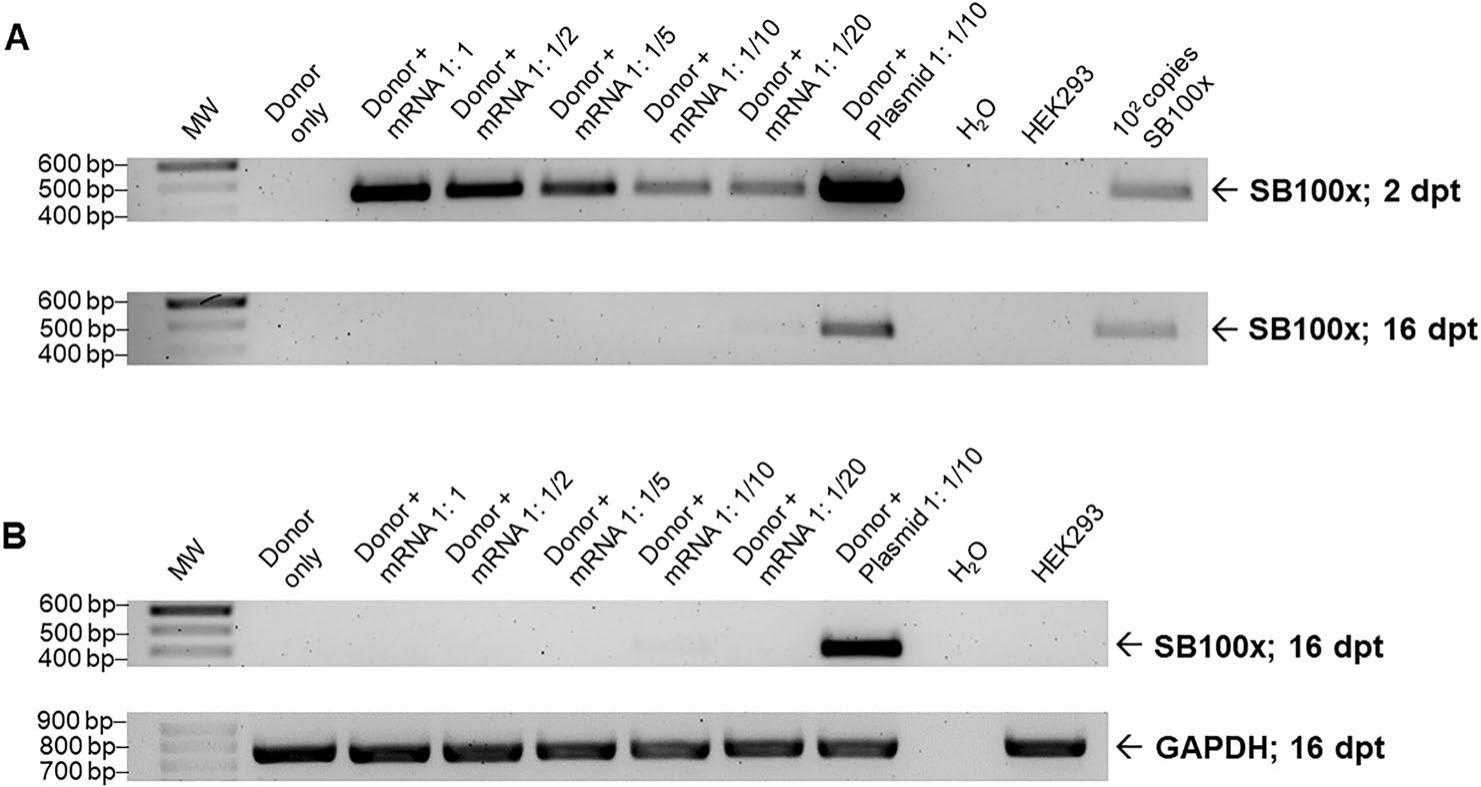
Detection of the transposase coding region in genomic DNA and RNA preparations of HEK293 cells after co-transfection. **A** Different ratios of donor vector SB-EGFP-IpW to SB100x-mRNA were transfected into HEK293 cells in duplicate and genomic DNA was isolated 2 and 16 days post transfection (dpt) for subsequent sensitive PCR analysis. In addition, co-transfections of the donor vector and the transposase plasmid CMV-SB100x and transfection with the donor vector exclusively were conducted. Transposase plasmid mixed with 60 ng genomic DNA of naïve HEK293 cells equivalent to 10,000 genomes served as positive control (10^2^ copies). Genomic DNA of naïve HEK293 cells and template-free samples (H_2_O) served as negative controls. **B** Detection of *SB100x* transcripts in HEK293 cells. RNA of cell pools was isolated 16 dpt. Equivalent volumes of synthesized cDNA were used for the amplification of DNA fragments of the *SB100x* coding sequence. Amplification of GAPDH served as an internal positive control. Naïve HEK293 cells and template-free samples (H_2_O) served as negative controls. Data shown are representative results of experiments conducted in duplicate

To prevent undesired integration of the transposase gene into the target genome, cDNA of *SB100x* was transcribed in vitro into SB100x-mRNA for subsequent co-transfection with the donor vector SB-EGFP-IpW (Fig. 1). Different ratios of SB100x-mRNA to donor vector were co-transfected into HEK293 cells to determine the optimal condition facilitating efficient transposition. Two and 16 days post transfection, genomic DNA was isolated and subjected to PCR using *SB100x*-specific oligonucleotides. As shown in Fig. 4A and two days post transfection, the transposase gene was detectable in all cell pools co-transfected with SB100x-mRNA or plasmid SB100x. Detected signal intensities decreased with higher dilution of transfected SB100x-mRNA. This indicated the contamination of SB100x-mRNA with the IVTRup-SB100x-derived amplicon DNA. However, after 16 days of cultivation under constant selection pressure, the transposase gene was exclusively still detectable in genomic DNA of cells co-transfected with plasmid SB100x. Cell pools transfected with SB100x-mRNA showed no longer a contamination with SB100x DNA at a sensitivity of detection of 10^2^ copies per 10,000 genomes. It could therefore be assumed, that the IVTRup-SB100x plasmid was detected at 2 days post transfection in its epichromosomal form and did not stably integrate into the cell genomes at high frequency.

In parallel and to examine transposase expression, RNA was isolated and transcribed in vitro into cDNA for subsequent PCR after 16 days of cultivation. Transposase gene expression was readily detected only in samples of cells co-transfected with plasmid SB100x. In contrast and underscoring the findings employing genomic PCR, no transposase transcripts were detected in cells transfected with SB100x-mRNA.

### FACS Analysis 16 Days Post Transfection Using SB100x-mRNA

**Fig. 5:**
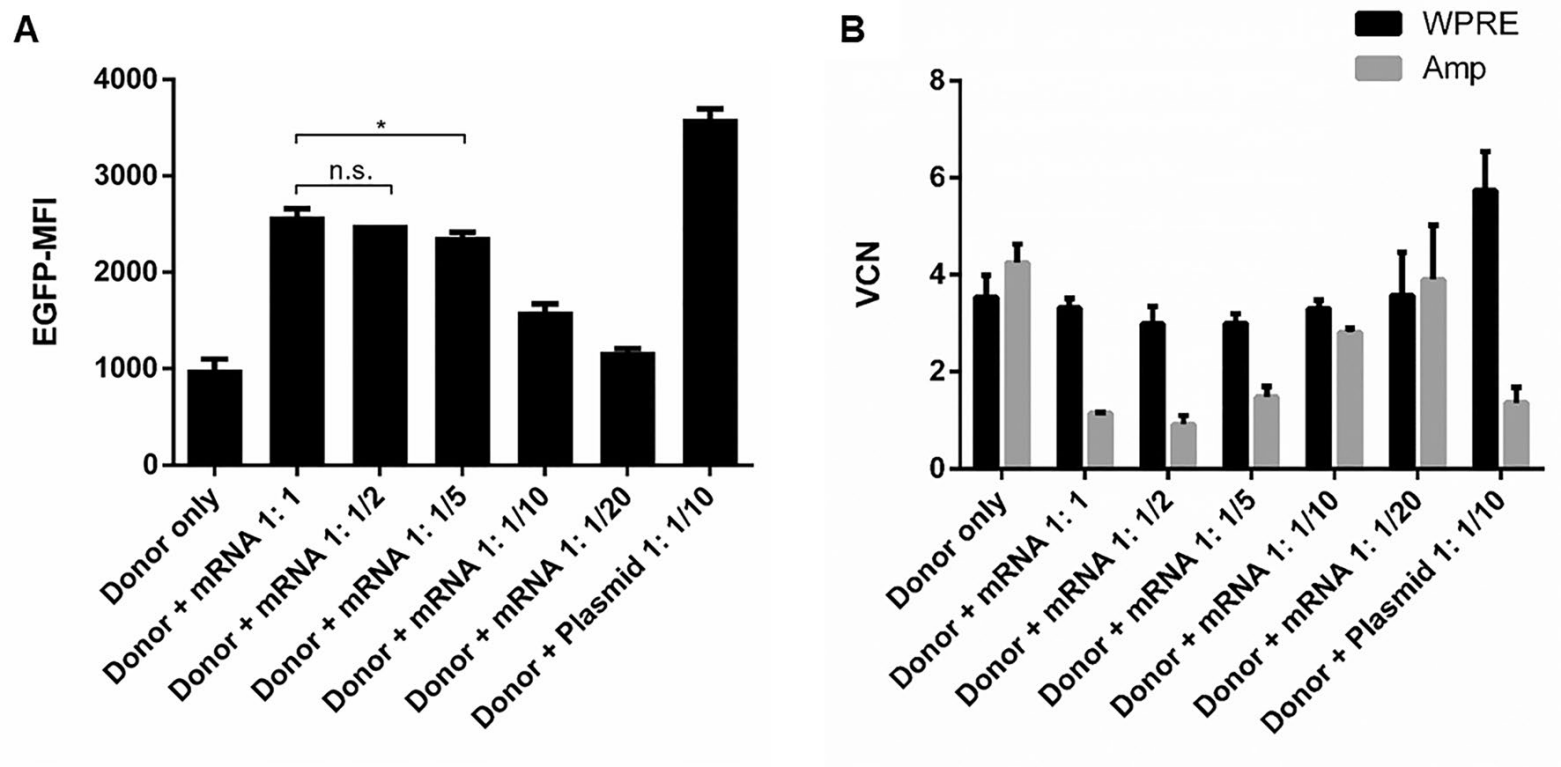
Flow cytometric analysis of HEK293 cells and determination of vector copy number (VCN) per genome from different cell pools 16 days post transfection after constant selection performed in duplicate. **A** HEK293 cells were co-transfected with different ratios of donor vector SB-EGFP-IpW to SB100x-mRNA and were analyzed for mean fluorescence intensity of EGFP expression (EGFP-MFI) employing flow cytometry. In addition, cells transfected with the donor vector only and co-transfected with the vector CMV-SB100x were analyzed. Mean values are indicated and error bars represent standard deviations. (*) A *P* value ≤ 0.05 is indicated for the ratios Donor + mRNA 1:1 and Donor + mRNA 1:1/5. The difference of mean EGFP-MFI values of ratios Donor + mRNA 1:1 and Donor + mRNA 1:1/2 is not statistically significant (n.s.; using the one-way ANOVA analysis). **B** Determination of VCN 16 days post transfection. Genomic DNA was isolated of HEK293 cell pools transfected with different amounts of SB100x-mRNA or transposase expression plasmid CMV-SB100x and subjected to Taqman®-qPCR using oligonucleotides allowing for the amplification of DNA fragments of WPRE in the donor vector and ampicillin (Amp^R^) present in the backbones of all constructs used. An additional primer/probe set amplifying the *ptbp2* gene was used as an internal reference. Mean values are shown and error bars represent standard deviations resulting from two independent experiments

To investigate the optimal ratio of donor vector to SB100x-mRNA for elevated transposition efficiencies resulting in high reporter gene expression levels, cells were subjected to flow cytometric analysis to determine EGFP mean fluorescence intensity (MFI) 16 days post transfection under constant selection pressure. As shown in Fig. 5A, cells transfected exclusively with the donor construct revealed the lowest MFI (987 ± 112) followed by the cell pools transfected with SB100x-mRNA at a ratio of 1:1/10 (1592 ± 79) and 1:1/20 (1175 ± 32). The highest MFI showed the cells transfected with plasmid-based transposase construct (3595 ± 104). Cells transfected with mRNA at a ratio of 1:1; 1:1/2 and 1:1/5 exhibited comparable and second highest mean fluorescence intensities (2583 ± 74; 2490 ± 0; 2367 ± 49).

### Assessment of Vector Copy Number

Vector copy numbers (VCNs) stably integrated in host cell genomes were examined 16 days post transfection employing TaqMan® qPCR. VCNs were determined using WPRE-specific primers probes binding within the donor vector expression cassette. PTBP2 primers served as internal reference. As shown in Fig. 5B, all cell pools transfected with SB100x-mRNA and cells transfected with the donor construct only reached a copy number of three copies or more of the donor vector. Co-transfection of the donor vector and plasmid-based transposase resulted in a mean integration of six transposon vector copies.

To investigate whether integrated VCNs were a consequence of stable donor plasmid transfection or transposition, qPCR using ampicillin-specific primers was conducted allowing for the detection of plasmid backbones of donor vector, transposase construct, and in some cases pUC18 used as fill-up DNA. Transfection with SB100x-mRNA with a ratio of 1:1 and 1:1/2 to donor vector SB-EGFP-IpW revealed the lowest amount of stably integrated plasmid with a mean plasmid integration of around 1 per cells (1.1 ± 0.0; 0.9 ± 0.2). Cells transfected with SB100x-mRNA and donor vector at a ratio of 1:1/5 as well as co-transfection with the plasmid CMV-SB100x yielded about two plasmid copy numbers (1.5 ± 0.2; 1.4 ± 0.3), respectively. Transfection of mRNA in a ratio of 1:1/10 and 1:1/20 and transfection with the donor vector only led to the integration of around three to four copies of ampicillin resistance genes as a result of undesired stable transfection.

## Discussion

Transposon vector technology has fostered the establishment of recombinant cell lines for the production of biologics, vaccine candidates, and viral vector particles [1]. However, when the transposase is expressed from plasmid DNA, the undesired integration as a result from stable transfection and sustained expression of the transposase gene may cause genetic instability of selected polyclonal recombinant cell lines due to ongoing transposition events.

We thus investigated the integration and expression of the transposase gene after co-transfection of a donor vector and a transposase construct followed by subsequent selection of three stable cell pools. HEK293 cells were co-transfected with the donor vector SB-VEGF-IpW and transposase expression vector CMV-SB100x in a ratio of 1:1/10. The ratio was previously optimized in our laboratory for efficient transposition in HEK293 cells resulting in high expression of the gene of interest. Two days post transfection, cells were subjected to expansion in the presence of increasing concentrations of puromycin and were cultivated until 48 days post transfection. Genomic DNA of recombinant cell pools was isolated at different time points and examined for transposase gene integration. Employing sensitive PCR, cell pools revealed *SB100x* gene integration over the entire period of 48 days with an approximate VCN of > 10^2^ per 10,000 genomes. The much higher VCN at day 7 and 10 can be attributed to high amounts of unintegrated epichromosomal plasmid shortly after transfection.

To investigate whether the integrated *SB100x* genes also mediated the expression of the respective *SB100x* transcripts, RNA of recombinant cells was isolated 17 and 48 days post transfection and subjected to RT-PCR analysis. Sustained transposase gene expression was readily detected at both time points. Signal intensity decreased from day 17 to day 48 most likely indicating gene silencing due to DNA methylation attracted by the bacteria-derived sequences in the plasmid backbone [23, 24].

To circumvent the use of transposon encoding plasmid DNA, we transcribed the *SB100x* DNA sequence in vitro into mRNA and co-transfected different ratios of donor vector to SB100x-mRNA with the transposon vector plasmid SB-EGFP-IpW into HEK293 cells in duplicate. To compensate for reduced mRNA amounts, samples were supplemented with the fill-up plasmid pUC18. Control cells were transfected with the donor vector—plus fill-up pUC18—and together with the transposase construct CMV-SB100x, respectively. Transgenic cells were continuously selected in the presence of puromycin. Genomic DNA of selected cell pools were again analyzed for the presence of the *SB100x* gene using sensitive PCR. The transposase gene was readily detected in all samples taken 2 days post transfection including the mRNA co-transfected cells. It is likely to assume that this was caused by a contamination with the PCR amplicon generated from parental construct IVTRup-SB100x used for in vitro transcription of the mRNA. The protocol used here should therefore in future extend the time for DNase I treatment and elevate the enzyme concentration to eliminate future contaminations. However, the contaminating PCR amplicon must mostly have remained epichromosomal since all samples of mRNA co-transfected cells were tested negative at 16 days post transfection. In contrast and yet again, cells co-transfected with the plasmid CMV-SB100x integrated transposase genes were readily detected. Using RT-PCR analysis, only these cells were also shown to express the transposase genes.

To assess efficient reporter gene expression and donor vector transposition, cell pools were also examined for EGFP expression and vector copy numbers 16 days post transfection employing FACS analysis and quantitative Taqman® qPCR, respectively. Cell pools co-transfected with transposase vector CMV-SB100x revealed the highest EGFP-MFI followed by cell pools transfected with SB100x-mRNA in a ratio of 1:1, 1:1/2, and 1:1/5. Cell pools co-transfected mRNA with a ratio of 1:1/10 and 1:1/20 revealed comparable mean fluorescence intensities to donor only transfected cell pools. This showed that reporter gene expression could be increased by transfecting more transposase mRNA but reached a maximum using a ratio of 1:1/5.

To examine whether high EGFP expression levels were a result of efficient transposition, Taqman® qPCR was performed using primers amplifying the mRNA-stabilizing element WPRE with in the transgene expression cassette of the donor vector and Amp^R^-specific primers able to amplify from the backbones of all plasmids used in this study, namely, SB-EGFP-IpW, CMV-SB100x, and pUC18. As expected from the highest expression level of EGFP, cells co-transfected with the donor vector and the CMV-SB100x expression plasmid revealed also the highest VCN of about 6 for the transposon vector, while only less than 2 copies of the plasmid backbones were detected indicating efficient transposition. The cell pool co-transfected with plasmid-based transposase generated copy numbers of about 6 copies per cell and is in accordance with the results of Kolacsek et al. [25].

Cells co-transfected with the donor vector and fill-up plasmid revealed lower VCN of around 4 for the donor vector, but even slightly higher amounts of integrated Amp^R^ genes demonstrating that the integrations resulted from stable transfection and due to the adjacent bacterial sequence were prone to gene silencing [7, 8, 26]. Similar patterns were observed in cells co-transfected with transposase RNA at ratios of 1:1/10 and 1:1/20, respectively. However, with the high amount of fill-up plasmid used here to compensate decreasing vector component DNA concentrations, it is very likely that a large proportion of plasmid backbone detected originated from pUC18 integrations. This would in turn mean that most transposon vector copies resulted from transposition events and would explain higher EGFP expression levels as compared to the control cells. Cells transfected with RNA to plasmid ratios 1:1, 1:1/2, and 1:1/5 revealed much lower amounts of backbone sequences ranging from 1 to 2 but similar donor vector copies of about 3 per genome, and thus mostly integrated by transposition. This supports the findings of comparable high expression the EGFP transgene.

The elevated copy numbers using CMV-SB100x were presumably a result of the prolonged expression of the transposase gene over some of days as compared to the utilization of transposase mRNA, which only last for a couple of hours [19]. We assume this could be compensated conducting first co-transfections of donor vector and transposase mRNA followed by repeated serial transfections of only the mRNA e.g., at two or three successive days. During this time period, donor plasmid should still be available in sufficient quantities in the cells to enable efficient transposition. In summary, we anticipate that the avoidance of undesired transposase gene integration into the host cell genome, and thus sustained expression, using transposase IVT mRNA transcripts will be utilized in future cell line development processes.
